# Is economic dependence on the husband a risk factor for intimate partner violence against female factory workers in Nepal?

**DOI:** 10.1186/s12905-017-0441-8

**Published:** 2017-09-13

**Authors:** Sunita Dhungel, Pabita Dhungel, Shalik Ram Dhital, Christiane Stock

**Affiliations:** 1 Unit for Health Promotion, University of Southern Denmark, Niels Bohrs Vej 9-10, 6700 Esbjerg, Denmark; 2 Institute of Medicine (IOM), Tribhuvan University, Maharajgunj, P.O. Box 1524, Kathmandu, Nepal; 3 Ministry of Health, National Health Education, Information and Communication Center (NHEICC), Teku, Kathmandu, Nepal

**Keywords:** Intimate partner violence, Socio-economic factors, Factory workers, Women, Nepal

## Abstract

**Background:**

Violence related injury is a serious public health issue all over the world. This study aims to assess the association between several socio-economic factors and intimate partner violence (IPV) in Nepal.

**Methods:**

A cross-sectional study was conducted among 236 women working in carpet and garment factories in Kathmandu, Nepal. Interviews were conducted to collect quantitative data on three forms of IPV, namely physical violence, psychological violence and sexual violence, as well as on a number of potentially associated factors.

**Results:**

Twenty-two percent of women experienced sexual IPV, 28% physical IPV and 35% psychological IPV at least once in the last 12 months. The variables independently associated with at least one form of IPV were: age of the woman >29 years [OR = 4.23, *p* = 0.025 for physical IPV; OR = 6.94, *p* = 0.008 for sexual IPV; OR = 3.42, *p* = 0.043 for psychological IPV], alcohol consumption of the husband [OR = 9.97, *p* < 0.001 for physical IPV; OR = 3.76, *p* = 0.004 for sexual IPV; OR = 4.85, p < 0.001 for psychological IPV], education of the husband above primary level [OR = 0.43, *p* = 0.013 for physical IPV; OR = 0.51, *p* = 0.033 for psychological IPV], and economic dependency of the woman on the husband [OR = 3.04, *p* = 0.021 for physical IPV; OR = 2.97, *p* = 0.008 for psychological IPV].

**Conclusions:**

This study identified various factors associated with IPV and showed that economic dependence of wives on their husband was among the most important ones. Thus, for the prevention of IPV against women, long term strategies aiming at livelihood and economic empowerment as well as independence of women would be suggested.

## Background

Intimate Partner Violence (IPV) against women is a serious public health concern and a human rights issue worldwide [1–3]. IPV refers to any behavior that causes physical psychological and sexual harm to those who are involved in the intimate relationship. Such kind of harms are due to physical aggression (hitting, kicking, beating or slapping), psychological abuse (humiliation, threatening or insulting) or sexual abuse (forced sexual intercourse or controlling inappropriately) [2]. Intimate partner violence is associated with certain characteristics of males, such as young age, low level of education, witnessing or experiencing violence as a child, harmful use of alcohol and drugs, personality disorders, and acceptance of violence [1]. IPV includes all verbal expressions, manners and actions that violate one’s physical body, sense of self-esteem, or sense of trust and happens regardless of age, ethnicity and country of origin [3]. The prevalence of IPV varies within communities, countries and regions reflecting that the violence against women is not inevitable throughout the world [4]. A study of the World Health Organization (WHO) in 2013 showed that 35% of women worldwide have experienced either physical and/or sexual intimate partner violence or non-partner sexual violence [5]. Other studies about the prevalence of IPV have shown that between 20% and 68% of women aged 15–49 years have experienced physical or sexual violence or both from the male intimate partner in their lifetime and it has been noted that there is scarcity of research with regard to male perpetration although the problem is enormous [5–7].

In Nepal, IPV against women is encouraged due to the male dominance social system which discriminates against women and is also connected to cultural factors that limit women’s choices to leave a violent marriage [7]. Women have lower accessibility to ownership, less employment opportunities and poorer economic background [8]. There are only few studies focusing on the conditions of violence among female factory workers from other household members and especially from their intimate partners [9–11]. The Nepal Demographic and Health Survey (NDHS) 2011 showed that one-third of ever-married women aged 15–49 reported having experienced emotional, physical, or sexual IPV at some point, and 17% reported having experienced one or more of these forms of violence in the past 12 months [9]. Another study regarding domestic violence among 1296 women aged 15 to 24 years in rural Nepal showed that overall more than half of young married women (52%) reported having ever experienced some type of violence from their husbands, nearly half (46%) reported sexual violence, one-fourth (25%) reported physical violence, and nearly one in five (20%) women reported ever experiencing both sexual and physical violence [12]. Factors associated with IPV were female illiteracy, low economic status, violent family history, the husband’s level of education, higher number of children, and lack of decision making autonomy [9]. In addition, in many women working in the factories in the capital city of Kathmandu, Nepal migrated from rural Nepal to the capital due to which they have experienced changes in their lifestyle and behaviors. The social and geographic isolation from their home and communities can lead to influencing their traditional values and behavioral norms and this is likely to increase unprotected sexual activities followed by extramarital affairs. Such kinds of activities may pose challenges to marital relationships and may also contribute to the root cause of IPV among female factory workers [13].

The risk for IPV in Nepal also seems dependent on various socio-economic factors. Most of the women from under-privileged castes and ethnic groups are at higher risk for IPV than women from higher castes [9]. In addition, the risk of violence on women has been shown to be higher for women who are economically dependent on their husband than for economically independent women [14].

In the Nepalese context, economic dependency of women on their husband is generally high as compared to the western part of the world [8], which makes it highly relevant to study the association between economic dependence and IPV in Nepal. There are no studies on IPV among women working in factories in Nepal, although the risk factors for IPV are very common in these groups [15]. Due to the relatively low salary for female factory workers this group of women still depend economically on their husband, although they are employed [11, 16]. The population of female factory workers was chosen for this study, because this population represents the urban-rural population with lower socioeconomic status and lower educational level in Nepal [11, 13]. Since IPV is a neglected issue in Nepal with low priority given by policy makers and planners and even in the civil society, there is a need to establish new knowledge for health planning in communities.

Therefore, this study aimed at estimating the prevalence of physical, sexual and psychological IPV and at studying demographic, educational, social and economic factors associated with IPV among married women in the reproductive age between 15 and 49 years working in factories in Kathmandu.

## Methods

### Data collection

This cross-sectional study used a standardized, closed ended questionnaire administered orally by interviewers to married women of reproductive age (15–49 years) working in factories in Kathmandu, Nepal. Four carpet and garment factories were included in the study, because the carpet and garment industry is traditionally employing mostly women. The factories were selected on basis of convenience. The study population comprised of only married women currently living with their husband and the sample size calculation was done with OpenEpi software [17]. The sample size of 236 was sufficient to estimate an IPV prevalence of 30% with a 95% confidence interval (CI) of +/− 3%. Convenience sampling technique was used to select the sampled individuals. Women whose husbands were working abroad and currently not living together with them were not included in the study. The study participants were informed about the study, that their participation was voluntary and that they can terminate the interview at any time. Verbal consent was given before starting the interview. As the data collection was done anonymously, the respondents were assured about the confidentiality of their data. No formal ethical clearance of the study protocol was done, as it is not mandatory for this type of study in Nepal. There were three interviewers involved in the data collection procedures. All interviewers were oriented about the application of questionnaires in the field and received instruction on how to conduct the interview and how to respect the sentiments and opinions of the respondents. All interviewers were females and were experienced in health research on sensitive issues in Nepalese communities. The questionnaire was administered by face-to-face interview method and answers from respondents (women) were noted immediately in the field.

The total number of respondents included in the study was 236, as it was the required sample size of the study. Data collection was done using purposive sampling technique, meaning that sampling was done until the number of data reached the required sample size i.e. 236. Four factories were included to reach the required sample size. None of the eligible women refused to participate in the study nor did not complete the interview resulting in a response rate of 100%.

### Questionnaire

The structured closed ended questionnaire was constructed by taking the reference from standard survey tools used by the Government of Nepal for the NDHS 2011 for collecting information about IPV at national level [18]. The questionnaire was initially developed in the English language and was later translated into Nepali language by taking care of the proper meaning of questions and not changing the original theme of the content. The translated Nepali version of the questionnaire was checked by a researcher who was fluent in both English and Nepali language. The tool was pretested in the similar setting to assess whether the questions are easy to understand, respondent friendly and to make necessary corrections according to the level of understanding of respondents.

The first part of the questionnaire included general characteristics of women such as their age and their age at first marriage (>20 years/20 years or above according to NDHS [9]). The women were also asked about the number of children (<2/2 or more) according to the Ministry of Health and Population of Nepal [19]). In addition, we asked whether their marriage had been arranged or was based on love. The participants were also asked about the level of education and that of their husband (Below or equal to primary level/Above primary level). Considering female literacy is lower than male literacy in Nepal [9], the educational status of women was categorized into Yes or No school attendance. We also asked for their monthly income, the sufficiency of their income for monthly expenses of the family (Yes/No), and whether they have capacity to make their own decisions on major household purchases (Yes/No) according to the NDHS Survey [9]. Economic dependence of woman was assessed by asking if the woman has to depend on their husband for the household expenditure during the past year (Yes/No). The respondents were further asked about the frequency of their own alcohol consumption as well as whether or not their husband drinks alcohol (Yes/No).

#### Measurement of IPV

Three forms of IPV were studied, namely physical violence, psychological violence and sexual violence. Physical violence included those acts causing physical harm such as hitting, beating, kicking, slapping, dragging or any form of physical injury. Psychological violence included those acts causing psychological harm such as insulting, threatening, humiliating or controlling on unnecessary activities and sexual violence, including forced sex. The time period for experiencing violence was during the last 12 months. The presence of any form of violence among those three types of violence was considered as the presence of IPV. The response was categorized binary as Yes and No.

Psychological violence was assessed by the question: ‘Did your husband ever insult you or make you feel bad about yourself?’ Physical violence was assessed by a single question: ‘Have you ever been hit, slapped, kicked or done anything else to physically hurt by your husband at times?’ Sexual violence was assessed by a question: ‘Has your husband ever forced you to have sex when you did not wish to do so?’ The responses for all forms of IPV were in Yes/No form and for the severity assessment, the response options were ‘often’, ‘sometimes’, and ‘not at all’. The participants were also asked about their knowledge about IPV against women with the question: ‘Do you know what intimate partner violence is?’

### Statistical analysis

Data was entered and analyzed in SPSS 22 for Windows. Chi-square tests were conducted to assess any association between the three forms of IPV and relevant socio-demographic factors. A significance level of *p* < 0.05 for the Pearson value of the Chi-Square test was taken as indicating significance. Only factors significant in the Chi-Square tests (data not shown) were used in the bivariate logistic regression models presented in Tables 2, 3 and 4. Multifactorial logistic regression analyses were performed to study factors associated with physical, sexual and psychological IPV as dependent variables. Odds ratios (OR) and 95% confidence intervals (CI) were calculated and presented for each single factor and also when adjusted for all other variables in the Table.

## Results

### Description of the sample

Table 1 shows the general characteristics of women participating in the study. The mean age of the women was 30.36 years (SD = 7.17). The highest percentage of women belonged to the age group 26–33 years (34%) or 19–25 years (33%) and the lowest percentage of women belonged to the group of 41–47 years (11%). The mean age of women at their first marriage was 19.88 years (SD = 1.94). Although the majority of women (59%) had their first marriage at the age 20–24 years, there was also a high percentage of women (39%) marrying at 15–19 years of age and only few respondents (2%) were married at 25–29 years of age. The average number of children of women included in the study was 1.82 (SD = 1.07). The majority of women (58%) had an arranged marriage. The information on educational status of women showed that the majority of women (63%) had attended school and the majority of the women’s husbands (58%) had education above primary level. Regarding knowledge about IPV, the majority of the women (62%) knew about the meaning of IPV. In terms of finances, most of the women (71%) had a monthly income of 6.000–7.000 Nepalese rupees and only a minority of the women (17%) was earning more than 10.000 Nepalese rupees (about 100 USD). About half of the women (52%) used to make a decision about major household purchase along with their husbands and only a minority (18%) made such decisions on their own without asking their husbands. Regarding alcohol consumption, more than half of the women’s husbands (60%) used to drink alcohol. Among those women who reported drinking alcohol, 29% drank alcohol once a week, 20% of them drank alcohol two to four times a week, and only 11% drank alcohol every day. About three quarters of the women (74%) were economically dependent on their husband.

**Table 1:** Characteristics of the study population

Variables	Mean (SD)	N (%)
Mean age 30.36 (7.17)		
Age category		
19–25		78 (33%)
26–33		79 (34%)
34–40		52 (22%)
41–47		27 (11%)
Mean age at first marriage 19.88 (1.94)		
Age at first marriage		
15–19		93 (39%)
20–24		139 (59%)
25–29		4 (2%)
Average number of children 1.82 (1.07)		
Marriage type		
Arranged marriage		137 (58%)
Love Marriage		99 (42%)
Education of women		
No		87 (37%)
Yes		149 (63%)
Education of husband		
Below or equal to primary level		96 (42%)
Above primary level		140 (58%)
Knowledge about IPV on women		
No		90 (38%)
Yes		146 (62%)
Monthly income of women		
< NRs 6000		52 (22%)
NRs 6000–10,000		167 (71%)
> NRs 10,000		17 (17%)
Decision on major household purchase		
Women		42 (18%)
Husband		71 (30%)
Both women and husband		123 (52%)
Alcohol consumption of the husband		
No		95 (40%)
Yes		141 (60%)
Frequency of alcohol consumption		
Never		95 (40%)
Once in a week		68 (29%)
2–4 times in a week		46 (20%)
Everyday		27 (11%)
Economic dependence on husband		
No		61 (26%)
Yes		175 (74%)
Total		*N* = 236

### Prevalence of IPV in the last 12 months

Psychological violence was the form of violence most often reported and 35% reported that they had experienced psychological IPV by their husbands. Women, who reported that they were hit, slapped, kicked or physically harmed by their husband (physical IPV), included 28% of the respondents and 22% had suffered from sexual IPV.

### Factors associated with physical IPV

Table 2 shows the results of logistic regression models for the association between general characteristics, social and economic factors with physical IPV. The age of women above 29 years was a risk factor for physical IPV even when adjusted [Adj OR = 4.23, CI = 1.20–14.88]. The adjusted odds of physical IPV was also higher among women having economic dependence on their husband than among women who didn’t have economic dependence [Adj OR = 3.04, CI = 1.18–7.80]. The odds of physical IPV was about 10 times higher in women whose husband had a habit of drinking alcohol than those women whose husbands didn’t drink alcohol [Adj OR = 9.97, CI = 3.66–27.11] even when adjusted. The husband’s education was a protective factor for physical IPV [Adj OR = 0.43, CI = 0.22–0.83]. Several of the other variables showed an association with physical IPV only when not adjusted for the other variables: Having more than two children, having a love marriage, a lower educational level of the women and lack of knowledge about IPV. Decision making capacity of women didn’t show any significant association with physical IPV.

**Table 2:** Logistic regression of the variables associated with physical IPV

Variables	Crude OR (95% CI)	*P*-value	Adj. OR^a^ (95%CI)	*P*-value
Age of women				
Below or equal to 29 years	1.00	<0.001	1.00	0.025
Above 29 years	5.94 (3.05–11.57)		4.23 (1.20–14.88)	
Parity				
Less than or equal to 2	1.00	<0.001	1.00	0.780
More than 2	3.54 (1.92–6.53)		1.16 (0.39–3.45)	
Marriage type				
Arranged marriage	1 .00	0.048	1.00	0.532
Love marriage	1.79 (1.01–3.18)		1.24 (0.63–2.48)	
Education of women				
No	1.00	<0.001	1.00	0.315
Yes	0.26 (0.14–0.47)		0.67 (0.31–1.46)	
Education of husband				
Below or equal to primary level	1.00	<0.001	1.00	0.013
Above primary level	0.24 (0.13–0.45)		0.43 (0.22–0.83)	
Knowledge about IPV on women				
No	1.00	0.006	1.00	0.780
Yes	0.44 (0.25–0.79)		1.68 (0.60–4.71)	
Economic dependence on husband				
No	1.00	<0.001	1.00	0.021
Yes	4.66 (1.89–11.46)		3.04 (1.18–7.80)	
Decision making capacity of women				
No	1.00	0.255	1.00	0.946
Yes	1.56 (0.72–3.36)		0.97 (0.40–2.33)	
Alcohol use of husband				
No	1.00	<0.001	1.00	<0.001
Yes	13.30 (5.14–34.84)		9.97 (3.66–27.11)	

^a^ Odds ratio adjusted for all other variables in the table

### Factors associated with sexual IPV

Table 3 shows that the age of the women had a strong association with sexual IPV. The adjusted odds of sexual IPV was 7 times higher in women more than 29 years than those women below or equal to 29 years of age [Adj OR = 6.94, CI = 1.65–29.14]. Also, the adjusted odds of sexual IPV was almost 4 times higher in women whose husbands drank alcohol than those women whose husband didn’t drink alcohol [Adj OR = 3.76, CI = 1.54–9.17].

**Table 3:** Logistic regression of the variables associated with sexual IPV

Variables	Crude OR (95% CI)	*P*-value	Adj. OR^a^ (95%CI)	*P*-value
Age of women				
Below or equal to 29 years	1.00	<0.001	1.00	0.008
Above 29 years	8.25 (3.67–18.54)		6.94 (1.65–29.14)	
Parity				
Less than or equal to 2	1.00	<0.001	1.00	0.591
More than 2	3.77 (1.96–7.23)		2.71 (0.97–7.54)	
Marriage type				
Arranged marriage	1.00	0.016	1.00	0.817
Love marriage	2.16 (1.15–4.06)		1.09 (0.53–2.25)	
Education of women				
No	1.00	<0.001	1.00	0.433
Yes	0.28 (0.15–0.53)		0.72 (0.32–1.64)	
Education of husband				
Below or equal to primary level	1.00	0.030	1.00	0.934
Above primary level	0.50 (0.27–0.94)		1.03 (0.50–2.11)	
Knowledge about IPV on women				
No	1.00	0.015	1.00	0.370
Yes	0.46 (0.25–0.86)		1.59 (0.58–4.34)	
Economic dependence on husband				
No	1.00	0.005	1.00	0.070
Yes	3.99 (1.51–10.58)		2.57 (0.93–7.14)	
Decision making capacity of women				
No	1.00	0.014	1.00	0.122
Yes	2.74 (1.23–6.09)		2.01 (0.83–4.85)	
Alcohol use of husband				
No	1.00	<0.001	1.00	0.004
Yes	5.70 (2.44–13.32)		3.76 (1.54–9.17)	

^a^ Odds ratio adjusted for all other variables in the table

Several other variables only showed an association with sexual IPV, but only when not adjusted for the other variables in the table: women having more than 2 children, those having a love marriage, those with a lower level of education, those with a lower level of education of their husband, those who were economically dependent on their husband, those who had lack of knowledge about IPV and those who had decision making capacity were more likely to have experienced sexual IPV. However, all these associations became insignificant after adjusting for the other variables in Table 3.

### Factors associated with psychological IPV

Table 4 shows that women whose age was more than 29 years had 3.42 times the adjusted odds of having psychological IPV than younger women [Adj OR = 3.42, CI = 1.04–11.23]. The education of the husband was a protective factor for psychological IPV even after adjustment [Adj OR = 0.51, CI = 0.27–0.94]. Women’s economic dependence on their husbands was a risk factor for psychological IPV, i.e. the adjusted odds of this violence in women who had economic dependence on their husbands was 3 times higher than the odds of women without this dependence [Adj OR = 2.97, CI = 1.33–6.61]. Alcohol use of the husband was a risk factor for psychological IPV. The odds of psychological IPV was about 5 times higher for women whose husbands used alcohol than those not drinking alcohol even after adjustment [Adj OR = 4.85, CI = 2.37–9.95].

**Table 4:** Logistic regression of the variables associated with psychological IPV

Variables	Crude OR (95% CI)	*P*-value	Adj. OR^a^ (95%CI)	*P*-value
Age of women				
Below or equal to 29 years	1.00	<0.001	1.00	0.043
Above 29 years	4.00 (2.24–7.12)		3.42 (1.04–11.23)	
Parity				
Less than or equal to 2	1.00	<0.001	1.00	0.090
More than 2	3.88 (2.14–7.05)		2.49 (0.87–7.20)	
Marriage type				
Arranged marriage	1.00	0.122	1.00	0.675
Love marriage	1.53 (0.89–2.64)		0.87 (0.46–1.66)	
Education of women				
No	1.00	<0.001	1.00	0.217
Yes	0.31 (0.18–0.54)		0.63 (0.30–1.31)	
Education of husband				
Below or equal to primary level	1.00	<0.001	1.00	0.033
Above primary level	0.31 (0.18–0.55)		0.51 (0.27–0.94)	
Knowledge about IPV on women				
No	1.00	0.007	1.00	0.960
Yes	0.46 (0.27–0.81)		1.02 (0.40–2.60)	
Economic dependence on husband				
No	1.00	<0.001	1.00	0.008
Yes	4.14(1.92–8.92)		2.97 (1.33–6.61)	
Decision making capacity of women				
No	1.00	0.100	1.00	0.537
Yes	1.94 (0.88–4.29)		1.31 (0.55–3.12)	
Alcohol use of husband				
No	1.00	<0.001	1.00	<0.001
Yes	6.04 (3.08–11.83)		4.85 (2.37–9.95)	

^a^ Odds ratio adjusted for all other variables in the table

Several other variables only showed an association with sexual IPV, when not adjusted for the other variables in the table: Women having more than 2 children, those who had a lower level of education and those who had a lack of knowledge about IPV were more likely to having experienced psychological IPV. However, all of these associations became insignificant after adjusting for the other variables in Table 4. Type of marriage and decision making capacity of women were neither in the unadjusted nor in the adjusted model significantly associated with psychological IPV.

## Discussion

The first aim of our study was to study the prevalence of three different forms of IPV among female factory workers aged 15–49 years in Kathmandu Nepal. It was found that of 22% of women reported having experienced sexual IPV, 28% reported physical IPV and 35% reported psychological IPV at least once in their last 12 months. The NDHS 2011, a national level survey, reported slightly lower figures with 14% of women aged 15–49 years reporting sexual abuse by their husband at least once in their lifetime, 23% of them reporting physical abuse, one thirds of them reporting physical, sexual or psychological abuse once in their lifetime [9]. However, the comparison of the data from NDHS with our study may not be fully justifiable because NDHS was the national level study comprising of the women from the general population of Nepal, whereas in our study the respondents were recruited from the population of women working in factories of Kathmandu. In addition, the NDHS asked for lifetime abuse, while we asked for experienced IPV during the last 12 months. However, one can carefully conclude that the prevalence of IPV seemed to be higher among factory workers as compared to the general population.

As our second aim, this study focused on a number of relevant factors potentially affecting IPV which included age of women, age of women at their first marriage, number of children (parity), marriage type, education of women, education of husbands, economic dependence of women on their husbands, decision making capacity of women, knowledge about IPV on women and alcohol use of the husband. All these factors except for age of women at first marriage were significantly associated with the different types of IPV, but for most of the factors the association disappeared when adjusting for the other factors. The variables that were independently associated with at least one form of IPV were: higher age of the woman, alcohol consumption of the husband, low education of the husband and economic dependency of the woman on the husband. Out of these four variables, two of them showed a consistent association with all three forms of IPV even when adjusted for all other factors: higher age of women and alcohol use of the husband. Women whose age was 29 years and over had 3–7 times higher odds of having experienced psychological, physical or sexual IPV than younger women. The IPV risk for older women may increase due to more marital quarrelling that may be followed by violence [20], or older women may be more willing to reveal IPV than younger women or perhaps are more capable of challenging their husband with resulting violence. In line with our findings, a multi-cultural cross-sectional study from the United Nations about IPV showed that the prevalence of IPV against women by their husbands increased with increasing age of women [6] and a study in Columbia showed similar results [21]. However, another study in Tansania found higher IPV against younger women [22] indicating that the IPV dependence on age of women may differ from country to country.

Also, alcohol consumption of the husband was a strong independent risk factor for all forms of IPV. Alcohol consumption among men in Nepal is very high in comparison with the alcohol use of women [23]. Our results are in line with other studies showing that men who drink alcohol are more likely to be violent against their wife [24, 25]. Since the study participants were female factory workers who resided inside the factories premises and were not allowed to drink alcohol, only alcohol consumption of the husband was included in the study. Our study also indicated that a husband’s education above primary level was a protective factor for all forms of IPV without adjusting for other variables, but after adjustment the association of husband’s education remained only significant for psychological IPV. In agreement with our findings a study in the United States about characteristics of men who perpetrate IPV showed that education of the husband beyond high school was a protective factor for IPV [26]. School attendance of women was also found to be a protective factor for all forms of violence in the study, but the association disappeared when adjusting for the other variables. Another study on factors associated with IPV in women conducted in Nepal confirmed that female illiteracy was associated with IPV [7]. It can be assumed that the negative association between female literacy and IPV disappeared when adjusted for education of the husband, because the educational level of husband and wife are often correlated. In principle, education both of males and females could serve as a protective factor against IPV, because it may improve the competency for non-violent conflict solving.

Economic dependence on the husband was found to be a risk factor for all forms of IPV in this study and after adjustment remained significant for physical and psychological IPV. Previous studies about the association between IPV and economic dependence of women on their husband also showed that the risk of violence against women was higher for women who were economically dependent on their husband [14]. In Nepal, the economic dependency of women on their husband is higher than in many western countries, which limits the possibility of women to separate from a violent husband and to live an independent life. Although our study was carried out in female factory workers who were able to earn some money on their own, they still have to depend on their husband because of the low salary paid to them [11, 16].

Several other factors showed some association with IPV, but only in the unadjusted models. E.g. women with an arranged marriage were less likely to report IPV. In Nepal, marriages that were arranged by their family are more frequent than love marriages and this type of marriage is also more secured by the families that arranged the marriage [25].

Knowledge of women about IPV was found to be the protective factor for all forms of IPV in the study, but only without adjusting for other variables. A previous study about IPV conducted in Nepal concluded that women’s decision making autonomy was a strong predictor of IPV on women [7]. However, the results from our study did not show any significant association between decision making of women and any form of IPV after adjustment. We also identified an association between parity and all forms of IPV and women having more than 2 children were at higher risk for all forms of IPV, but when adjusted for other factors the associations disappeared. Another study about parity and IPV in the United States explored that each additional pregnancy was associated with 10% greater odds of IPV [26]. We explain the lack of significant associations regarding the factors above in the adjusted models as well as the differences compared to other research as we adjusted our analysis for more factors than most of the other studies that found significant associations.

We also found that age at first marriage was not associated with any form of IPV and therefore the variable was not included in the logistic models. This is in line with another study, which describes causes of IPV showing that age at marriage was not associated with IPV [20].

Our study has limitations and it is crucial to note that the data collection was cross-sectional and no conclusions regarding causal relationships can be drawn. The study population is also limited to factory workers in Kathmandu and the results may be different in rural areas or in other professional sectors. However, the response rate was extremely high which limits a potential selection bias towards only excluding women who may be absent from work due to IPV. Our study also relied on self-reported data and due to the fact that IPV and the associated factors are a sensitive issue, we cannot rule out potential under-reporting of IPV.

## Conclusions

In summary, we conclude that the prevalence of IPV is high in this group of women and that the odds of IPV was higher in women of higher age, for those with alcohol consumption of the husband, with low education of the husband and with economic dependency on the husband. Future research should identify underlying reasons for the high level of IPV.

The findings from this study are important in order to make Nepalese women aware of the problem and to enable advocacy about IPV. A holistic approach is essential to gain family and community trust as well as support for women at the micro, meso and macro levels for the prevention and control of IPV issues in Nepal [27]. The results of our study can be used to develop preventive programs. Such programs should aim at protecting women, including behavioral and educational programs for husbands and wives, as well as policy measures to reduce the underlying risk factors such as poverty and illiteracy. Strategies aiming at livelihood and economic empowerment and at increasing the economic independence of women would be suggested for the long-term prevention of IPV against women.
